# *Helicobacter pylori* Infection and Eye Diseases: A Systematic Review

**DOI:** 10.1097/MD.0000000000000216

**Published:** 2014-12-02

**Authors:** Sergio Claudio Saccà, Aldo Vagge, Alessandra Pulliero, Alberto Izzotti

**Affiliations:** From the IRCCS Azienda Ospedaliera Universitaria San Martino – IST Department of Head/Neck Pathologies, St Martino Hospital, Ophthalmology Unit, 16132 Genoa, Italy (SCS); Department of Neurosciences, Ophthalmology and Genetics, University of Genoa, Eye Clinic, 16132 Genoa, Italy (AV); Department of Health Sciences, University of Genoa, 16132 Genoa, Italy (AP, AI); Mutagenesis Unit, IRCCS Azienda Ospedaliera Universitaria San Martino – IST, National Institute for Cancer Research, 16132 Genoa, Italy (AI).

## Abstract

The connection between *Helicobacter pylori* (Hp) infection and eye diseases has been increasingly reported in the literature and in active research. The implication of this bacterium in chronic eye diseases, such as blepharitis, glaucoma, central serous chorioretinopathy and others, has been hypothesized. Although the mechanisms by which this association occurs are currently unknown, this review describes shared pathogenetic mechanisms in an attempt to identify a lowest common denominator between eye diseases and Hp infection.

The aim of this review is to assess whether different studies could be compared and to establish whether or not Hp infection and Eye diseases share common pathogenetic aspects. In particular, it has been focused on oxidative damage as a possible link between these pathologies.

Text word search in Medline from 1998 to July 2014.

152 studies were included in our review.

Were taken into considerations only studies that related eye diseases more frequent and/or known.

Likely oxidative stress plays a key role. All of the diseases studied seem to follow a common pattern that implicates a cellular response correlated with a sublethal dose of oxidative stress. These alterations seem to be shared by both Hp infections and ocular diseases and include the following: decline in mitochondrial function, increases in the rate of reactive oxygen species production, accumulation of mitochondrial DNA mutations, increases in the levels of oxidative damage to DNA, proteins and lipids, and decreases in the capacity to degrade oxidatively damaged proteins and other macromolecules. This cascade of events appears to repeat itself in different diseases, regardless of the identity of the affected tissue. The trabecular meshwork, conjunctiva, and retina can each show how oxidative stress may acts as a common disease effector as the Helicobacter infection spreads, supported by the increased oxidative damage and other inflammation.

## INTRODUCTION

*Helicobacter pylori* (Hp) is a major pathogen that is etiologically associated with gastritis, peptic ulcer disease, gastric cancer and primary gastric lymphoma. Worldwide, it is one of the most common chronic infections, although the exact mode of Hp transmission remains controversial. Transmission during transit disorders of the gastrointestinal tract has been suggested, although there is no evidence to date of its transmission during outbreaks of gastroenteritis.^1,2^ The host response to Hp precipitates the induction of damage to the gastric epithelium and therefore plays an integral role in Hp pathogenesis. Therefore, the Hp infection is acquired by oral ingestion of the bacterium and is mainly transmitted within families in early childhood.^3^ However, it is very difficult to identify the initial site of the Hp infection. Some authors have considered the role of Hp infections with eye diseases, including some afflictions as widespread as glaucoma and others as uncommon as mucosa-associated lymphoid tissue (MALT) lymphoma. Despite the repeated implication of Hp in the etiology of eye diseases, we still do not know the real of how Hp begins to target the eye. Mindel and Rosenberg, in 1997, for the first time, described Hp and ocular pathology.^4^ There are common risk factors that can be evident in several diseases. For instance, genome stability is essential for maintaining cellular and organism homeostasis, but it is subject to many discussions. One ubiquitous threat is from a class of compounds known as reactive oxygen species (ROS), which can indiscriminately react with many cellular biomolecules including proteins, lipids, and DNA to produce a variety of oxidative lesions. The human eye is constantly exposed to sunlight and artificial lighting. Exogenous sources of ROS such as UV light, visible light, ionizing radiation, chemotherapeutics, and environmental toxins may contribute to the oxidative damage in the tissues.^5^ The aging eye also appears to be at considerable risk from oxidative stress. Mitochondria are the major target of ROS, and alterations in the efficiency of mitochondrial respiration resulting in superoxide production. This precedes subsequent reactions that form potentially dangerous ROS species such as the hydroxyl radical, hydrogen peroxide and peroxynitrite. Therefore, during the life ROS-induced damage in the eye may consist of oxidation of proteins and DNA damage occurs in the various tissues of the eye. The ROS may be the *trait d’union* between eye diseases and infection by HP. The purpose of this review is to investigate the relationships between the eye and Hp infections and emphasize the common elements among the various diseases of the eye and the Hp infection and emphasize the elements that are shared between the different diseases of the eye and the Hp infection which are the possible common denominator of their pathogenesis.

## METHODS

We performed a literature search consisting of a text word search in Medline from 1998 to July 2014. Articles dealing with pathogenetic aspects of HP infection, eye disease, and oxidative damage and stress were selected and reviewed. The papers were sought in two databases Pub Med and Science Direct. The combinations of words that we used were: “Helicobacter AND eye” papers found: 82 (37); “Helicobacter AND pathogenesis AND eye” papers found: 76 (46); Helicobacter pylori infection AND pathogenesis AND oxidative stress“ papers found: 131 (77); “Eye AND pathogenesis AND oxidative stress“ papers found: 1557 (157). We reviewed only papers written in English. The number of papers actually reviewed is shown in brackets: all abstracts were read and, if subject was compatible with our article, the paper was reviewed in detail.

This article is a review, so it was not necessary to ask for the approval of this study to the ethics committee of the IRCCS San Martino University Hospital - IST.

### Helicobacter and Blepharitis

**FIGURE 1:**
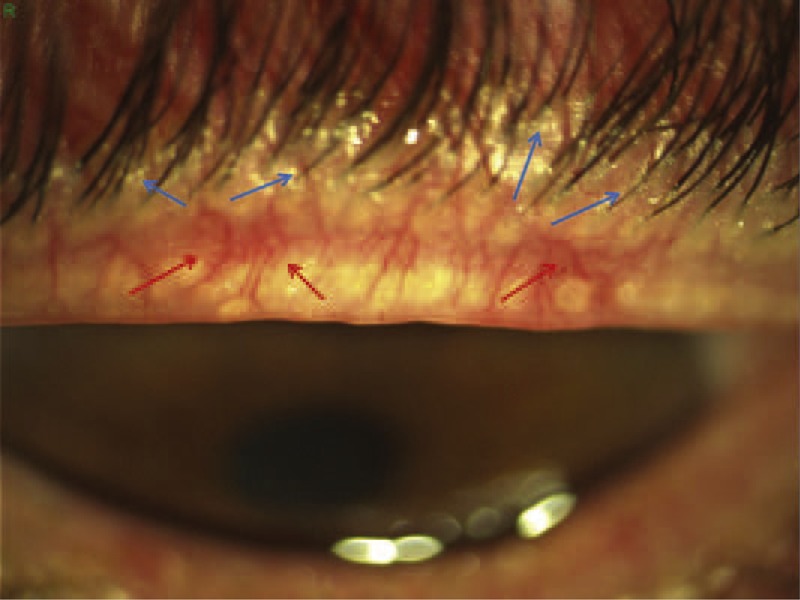
Anterior blepharitis of a mixed type of the upper eyelid. This shows a squamous blepharitis-hyperemic. Very evident the presence of collarettes, meibomian secretion around eyelashes (blue arrows) and telangiectasia and very hyperemic vessels of the lid margin (red arrows). Although it is unknown the role of Hp infection in this type of pathology, its eradication leads to an improvement in both subjective and objective symptoms and can, especially in the young, alleviate blepharitis.

Blepharitis is a common condition where the eyelids are inflamed, with oily particles and bacteria coating the eyelid margin near the base of the eyelashes. The underlying causes of blepharitis are not completely understood, it can be associated with a bacterial eye infection, dry eyes symptoms and certain types of skin conditions such as acne rosacea. From a dermatological aspect, a diagnosis of blepharitis is done if one or more lesions is observed on the eyelid margin in association with a non-granulomatous inflammatory reaction. This lesion is classified as rosacea blepharitis if the following were present: flushing; persistent erythema with scattered telangiectasia, papules and pustule; sprays of vessels, especially on the nose or cheeks; or phymas. Seborrheic blepharitis is diagnosed in the presence of the following signs: greasy-looking scales and/or crusts; increased redness and cutaneous color variability; or clinical patterns on the trunk, scalp, and face (including lids). Mixed type of dermatitis or blepharitis is diagnosed if one or more lesions fulfilling the criteria of both of the classification systems previously described were observed.^6^ (Figure 1) Rosacea is considered in conjunction with diseases of the anterior segment, such as blepharitis, nodular conjunctivitis, episcleritis and painful marginal infiltrates keratitis. It is interesting to underline that digestive troubles are more connected to rosacea than to seborrhea,^7^ although the two pathological conditions are closely related. According to Boni R., “Diseases of Seborrheic origin include rosacea, acne, gram-negative folliculitis, demodex-folliculorum, perioral dermatitis as well as Seborrheic dermatitis.”^8^

Hp is the primary cause of gastritis and a major contributor to peptic ulcer disease. There have been several investigations suggesting a possible etiologic role of Hp in rosacea.^9^ The link between seborrhea and Hp appears to be more uncertain, although it has been reported by other authors.^10^ Moreover, Seborrheic dermatitis may be observed in conjunction with other skin diseases, such as rosacea, blepharitis and ocular rosacea, and with acne vulgaris.^11^ Thus, it can therefore be claimed, although with some uncertainty, that digestive troubles may be correlated, or at least associated, with the presence of blepharitis. According to this hypothesis, another study was investigate the relationship between blepharitis and Hp.^6^ Although, possible sources of error must be considered when defining the association of two highly prevalent conditions, the data seem to validate an association between Hp infection and blepharitis. However, this association may still not be indicative of a causal association.^12^ Therefore, given that blepharitis and Hp infection are wide spread, it remains difficult to know whether this prevalence is real or rather random. Furthermore, several investigations have suggested a possible etiological role for Hp in rosacea,^13^ as the prevalence of Hp infection in patients with rosacea is higher than that in control subjects^13,14^ and Hp eradication treatment reduces the severity of rosacea.^14,15^ Overall, the relationship between blepharitis and Hp infection is not influenced by clinical appearance or degree^16^; it seems that their only common factor is chronic inflammation of the eyelid and gastrointestinal tract. Indeed, gastric epithelial cells release cytokines, such as interleukins, which act as proinflammatory stimuli, promoting the release of the other cytokines and contributing to the inflammatory state, in combination with the histamine from mast cell degranulation.^17^ Cross-mimicry mechanisms between bacterial and extradigestive antigens could affect extradigestive organs.^18,19^ Free radical and lipid peroxide generations are crucial to the attained events in inflammation; thus, Hp can increase the serum or tissue levels of nitric oxide,^20^ inducing vasodilatation, inflammation, and immune modulation. The effectiveness of the therapy is certainly connected with the lid inflammation, but it is not possible to know whether rosacea plays a greater role than the other blepharitis types.^13,15^ Chronic blepharitis is one of the most difficult ocular diseases to treat, and we do not know whether the antibiotics that treat the digestive disturbance have a secondary effect on the blepharitis. Thus, the Hp eradication therapy treats the infection at the same time as acts on the state and the flora of the eyelids. This is indeed plausible, as antibiotics may have local effects, in addition to systemic effects. Thus, these drugs may act on the conjunctival and lid bacteria, as well as on the Hp. In some cases, it has been demonstrated that the associations among chronic blepharitis, eyelid meibomian gland lipids and the microflora reveal important relationships between these lipids and chronic blepharitis disease states. Antibiotics, such as tetracycline, inhibit lipase activity, thereby decreasing the release of noxious free fatty acids.^21^ Dougherty et al^22^ have shown that tetracycline results in decreased bacteriological lipase activity in vitro.^23^ Therefore, it is reasonable to suppose that antibiotics act upon blepharitis. The cause of rosacea remains unknown, even if the associations between rosacea and certain digestive diseases, such as gastritis, hypochlorhydria, or a number of jejunal mucosal abnormalities, are well established.^16^ Among many theories, the role of Hp has often been a subject of investigation.^24^ Different studies have presented conflicting results. The mainstay treatments of ocular rosacea are topical metronidazole and oral tetracyclines, administered over several months. Furthermore, topical metronidazole, which is effective for stage I and stage II rosacea and avoids the toxicity of systemic treatment, is regarded as a first-line therapy.^25^ Rosacea responds well to oral antibiotics,^26^ and its systemic treatment includes metronidazole, which was used in this clinical study. Therefore, is difficult to understand how and whether there can be a direct infection of the eyelid with the Hp. Although Hp has been found in the mouth,^27^ the presence of Hp in a human oral cavity should be considered transient and independent of its oral status,^28^ but there is as of yet no research showing a direct causal relationship between Hp infection and diseases. Finally, there are currently no other studies seeking to understand the relationship between blepharitis and Hp.

### Helicobacter and Glaucoma

Glaucoma affects more than 70 million people worldwide.^29^ Glaucoma is a progressive optic neuropathy characterized by a modification of optic nerve head and visual field damage, which result from the loss of retinal ganglion cells by apoptosis.^30^ Moreover, in high tension glaucoma oxidative stress is the “primum movens" that affects trabecular meshwork (TM),^31^ particularly its endothelial cells.^32^ In these develops a real mitochondriopaty. Indeed, mitochondria produce up to 90% of required cellular energy and play a crucial role in mediated cell death through apoptotic pathways.^33^ Damage accrued by the mitochondrial genome (mtgenome) is associated with increased cellular stress and organelle dysfunction.^34^ TM cells of patients with primary open angle glaucoma (POAG) have lower levels of ATP, as their functionality is endangered by an intrinsic mitochondrial complex I defect that leaves these cells mitochondrial respiratory chain-deficient.^35^ Mitochondrial DNA (mtDNA) deletion is dramatically increased in the TM of POAG patients, compared to controls, and the ratio between mtDNA and nuclear DNA is decreased; additionally, the amount of nuclear DNA per mg wet tissue is decreased,^36^ confirming that mitochondrial damage is severe in the TM of POAG patients. The trabecular meshwork altering both motility and cytoarchitecture, inducing cells die by apoptosis, losing barrier functions and altering the aqueous humor outflow. This is the reason intraocular pressure (IOP) increase occurs during glaucoma.^32^ Under normal physiological conditions, approximately 1%–5% of the oxygen consumed by mitochondria is converted to ROS, including superoxide anions, hydrogen peroxide, and hydroxyl radicals.^37^ Mitochondrial respiratory function declines with age,^38,39^ which increases the production of ROS and free radicals in mitochondria. Further, ROS and oxidants can function as intracellular signaling molecules, conditioning cell death or survival.^40^ The wide spectrum of alterations in aged individuals and senescent cells is correlated with the cellular response to a sublethal dose of oxidative stress. These alterations and responses include the following: (1) decline in mitochondrial respiratory function; (2) increase in the rate of production of ROS; (3) accumulation of mitochondrial DNA (mtDNA) mutations; (4) increase in the levels of oxidative damage to DNA, proteins and lipids; and (5) decrease in the capacities of degradation in oxidatively damaged proteins and other macromolecules.^41^ Responses to oxidative stress and their subsequent interactions in tissues result in deleterious effects on cellular functions, which culminate in aging and degenerative diseases. Oxidative modification and mutation of mtDNA occur with great ease, and the extent of such alterations of mitochondrial DNA increases exponentially with age. Oxidative modification in mtDNA is much more extensive than in nuclear DNA.^42,43^ Age-related alterations in the respiratory enzymes not only decrease ATP synthesis but also enhance the production of ROS by increasing electron leakage in the respiratory chain. With the accumulation of genetic defects in mechanisms of mitochondrial energy production, the issue of neuronal susceptibility to damage as a function of aging becomes important.^44^ Damage to mtDNA induces alterations to the polypeptides encoded by the mtDNA in the respiratory complexes, resulting in consequent decreases in electron transfer, further production of ROS and a vicious circle of oxidative stress and energetic decline. This deficiency in mitochondrial capacity is considered the cause of aging and age-related degenerative diseases.^45^ Markers of cellular senescence are found in the TM of patients with POAG to a much greater degree than in age-matched controls,^46^ besides, mitochondria provide a gene-environment interaction between environment and our genes.^47^ The delayed-onset and progressive course of age-related diseases are based on the accumulation of somatic mutations in the mtDNAs of postmitotic tissues. The variations in individual and regional predispositions to degenerative diseases and cancer may result from the interaction of modern dietary caloric intake and ancient mitochondrial genetic polymorphisms.^47^ As mentioned above ROS are most likely responsible for TM malfunctions, which lead to IOP increases.^48^ Indeed, during the course of POAG, the most severe TM alterations occur in the layers that are in closest contact with the aqueous humor of the anterior chamber,^49^ whose cells are exposed to relatively high hydrogen peroxide concentrations.^50^ Extensive and prolonged oxidative stress in vivo results in reduced TM cell adhesion, cell loss, and compromised TM integrity.^51^ The peculiar sensitivity of the TM to oxidative stress is consistent with the damage selectively induced, which triggers glaucoma pathogenic cascade.^52^ The oxidative damage detected in TM^53^ could not explain why patients with glaucoma exhibited low levels of circulating glutathione, suggesting a general compromise of the antioxidative defenses.^54^ Furthermore, the statistically significant correlations between TM oxidative damage, and IOP increases was observed in POAG^55^ cannot be explained by assuming this relationship as a result of drug administration. Moreover, the increased expression and activity of nitric oxide synthase in the TM of POAG patients are proportional to the visual field defect and could lead to increased nitrotyrosine levels, which in turn may serve as markers of oxidative stress in the progression of TM cell death in POAG.^56^ Antioxidant proteins are downregulated in the increased of nitric oxide synthase 2 and in the presence of other proteins that, under physiological conditions, are segregated inside cells into functional mitochondria.^36^ Under POAG pathological conditions, mitochondrial proteins can be detected in AH, thus demonstrating the occurrence of cell and mitochondrial damage and destruction.^57^ These data support the hypothesis that, as in many neurodegenerative diseases, there is mitochondrial dysfunction in glaucoma. Mitochondrial damage triggers intracellular calcium release and the activation of apoptosis through the intrinsic activation pathways.

**FIGURE 2:**
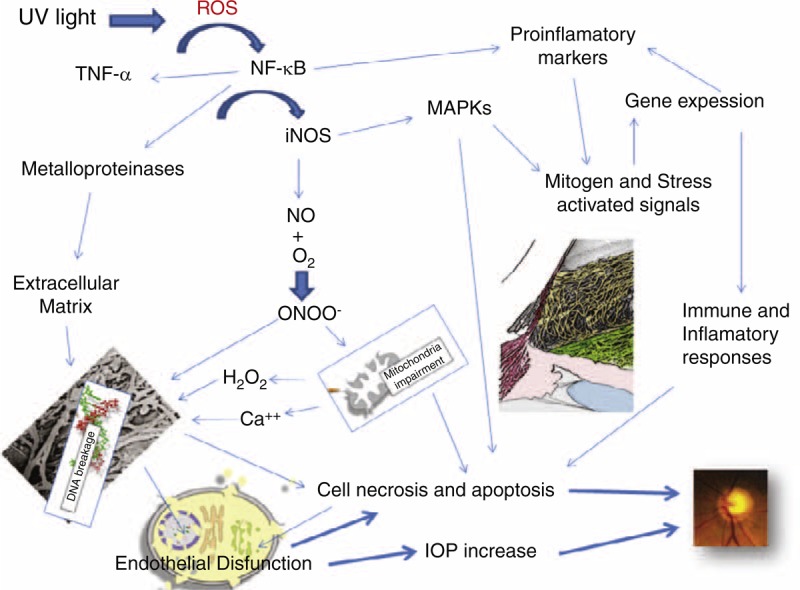
Glaucoma in oxidative stress plays a key role in mitochondrial dysfunction occurs and consequently the endothelial cells of the trabecular meshwork are not working as they should. There is an alteration of the extracellular matrix is accompanied by the activation of several metabolic pathways that result in an alteration of gene expression, an activation of inflammation and the immune response. This determines the malfunction of the trabecular meshwork and consequently the intraocular pressure increase. All this triggers the apoptosis of retinal ganglion cells.

Apoptosis occurring in ocular tissues during POAG is induced by a variety of mechanisms, primarily including mitochondrial damage but also inflammation, vascular dysregulation, and hypoxia.^58^ Overall, several proteome alterations confirm the occurrence of oxidative stress in the anterior chambers of POAG patients.^57^ In particular, the antioxidant enzymes superoxide dismutases 1/2 and glutathione S transferees 1 were significantly lower in POAG patients than in controls, while the pro-oxidant enzymes, nitric oxide synthesize 2 and glutamate ammonia ligase, were significantly higher in POAG patients than in controls^59^ (Figure 2).

**FIGURE 3:**
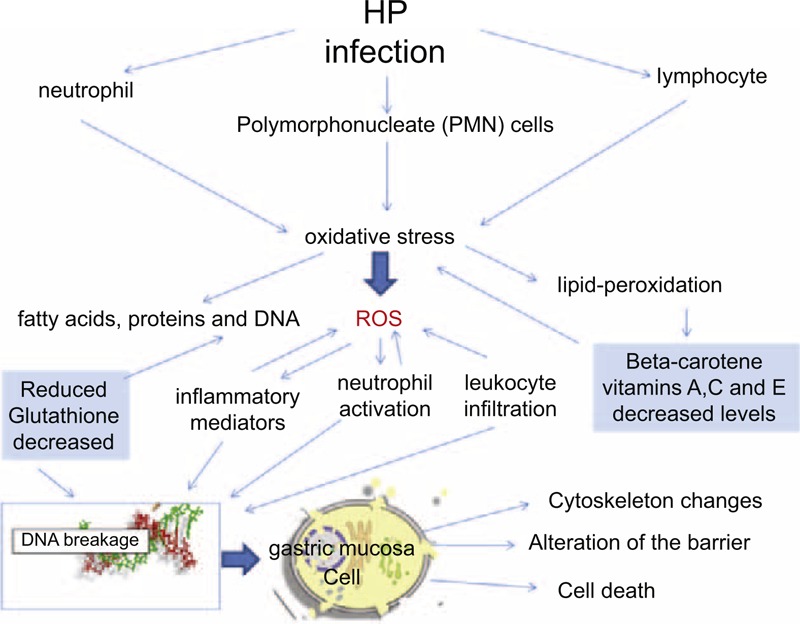
Sequence of the pathogenesis of Hp infection that increases the production of ROS and inflammatory cytokines, sees the recruitment of white blood cells in the presence of a decrease of antioxidant defenses leads to the alteration of gastric mucosa cells and thus of the gastric mucosal barrier.

Hp infection has been associated with glaucoma.^60–62^ A positive association between Hp infection and ROS production was first demonstrated in 1994.^63^ Recently, Hp bacteria were identified in the trabeculum and iris specimens from patients who underwent trabeculectomy for POAG.^64^ Furthermore, Hp infection locally induces a chronic inflammatory status consisting of polymorphonuclear neutrophil and lymphocyte recruitment at the infection site^65^ (Figure 3).

Oxidative stress is exacerbated by both relative deficiencies of glutathione and of vitamins A, C, and E.^66,67^ In addition, the recruitment of neutrophils and the release of a variety chemoattractants/inflammatory mediators triggers an intense leukocyte infiltration of the gastric mucosa, which can cause tissue damage in the absence of antioxidants.^68^ This Gram-negative bacterium activates multiple oncogenic pathways in epithelial cells, including NF-kappaB, and induces epigenetic alterations, such as DNA methylation and histone modification, which play critical roles in oncogenic transformation.^69^

Hp can be classified into two different classes based on its ability to produce cytotoxins, such as *CagA* (cytotoxin-associated gene A) and *VacA* (Vacuolating-associated gene A).^70^ Inside the cells, VacA can target mitochondria, leading, at least in some cases, to the release of cytochrome *c* and apoptosis.^71,72^ Even if the actual sequence of events is unknown, a potential mechanism is that Hp infection reaches the mitochondrion from the endosomal compartment after accumulation, allowing a specific and direct interaction between the endosomal membranes and the mitochondrial outer membranes.^73^ The chain of events leading to the apoptosis of endothelial cells (in glaucoma) and of gastric mucosa cells (in gastric Hp infection) are oxidative stress with mitochondrial involvement; this is similar in both diseases. Another similarity between the two diseases is given by the alteration of the barrier function mediated by the signaling of the Rho family of GTPases.^57^ The rho-kinase pathway appears to mediate TM cell responses to cyclic mechanical stress.^74^ During the course of glaucoma, TM endothelial cell alterations arise, leading to increased pressure to other molecular events that then translate into the clinical apoptosis of ganglion cells and, thus, to the visual field detriment.^58^ Hp changes based on epithelial cell signaling and polarity, which can explain the pathogenesis of the carcinoma.^75^ Finally, it is worth noting the potential contributions of an Hp infection to vascular injury. Indeed, certain pathogens, such as Hp, can increase the synthesis of tissue factors, cell-surface thrombin expression, platelet adherence, and the expression of adhesion molecules, cytokines, and growth factors, even while decreasing prostacyclin release^76,77^ and causing endothelial cell injury.^78^ Endothelial dysfunction may be one of the underlying mechanisms by which multiple intracellular pathogens actually contribute to these early processes that lead to the development of atherosclerosis and to its progression to multi-vessel disease.^79^ Hp infection stimulates the production of the proinflammatory cytokines associated with the development of atherosclerosis,^80^ which arise cellular oxidative stress and endothelial dysfunction^81^; furthermore, Hp eradication can improve endothelial dysfunction.^82^ The high-tension glaucoma depends of the endothelial cell dysfunction in the TM.^58^ The anterior chamber of the eye is a real vessel^83^ that behaves as if it were a vase, expressing all proteins that act as early markers of plaque atherosclerosis.^84^ Still, we do not really know whether the prevalence of glaucoma is significantly different in Hp-infected patients from that in non-infected subjects. There are no epidemiological studies that have demonstrated the possible ethnic similarities and/or diversities regarding the associations between Hp and glaucoma among different countries.^85^ Therefore, Hp infection was associated with risk for normal tension glaucoma (NTG). Indeed, the retinal ganglion cells may be damaged in eyes within normal intraocular pressure in because the site of injury related with Hp infection may be not only trabecular meshwork but also the retinal ganglion cell itself. Probably, It due by decreasing ocular blood flow, secreting toxic materials, and causing antibody-induced apoptosis attributed to inflammation in the retrobulbar area, although the exact pathophysiology is still unclear.^86^

### *Helicobacter pylori* and Central Serous Chorioretinopathy

**FIGURE 4:**
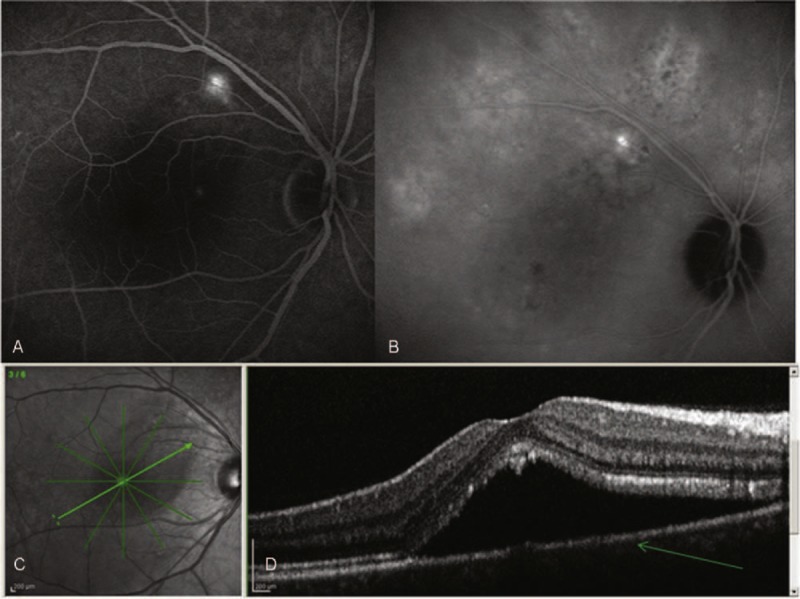
(A) Fluoroangiography of right eye; it shows a central serous chorioretinopathy which highlights the leakage (white) in the retinal pigment epithelium with pooling of the dye in the subretinal space. (B) Indocyanine green angiography shows an area with a late iperfluorescent likeage that suggests as an etiologic factor the choroidal hyperpermeability. (C) The optical coherence tomography (OCT) allows us a qualitative and quantitative assessment of the disease. (D) OCT cross-sectional image of a fovea with central serous chorioretinopathy: The detachment of the neurosensory retina is revealed as optically empty space within the retina. The retinal pigment epithelium remains attached to the choroid and it is evident by OCT as a thin layer (arrow). The retinal layers are intact but detached in the photoreceptors are thicker and a higher reflectivity.

Central serous chorioretinopathy (CSCR) is characterized by an acute, serous detachment of the sensory retina in the macular region by idiopathic breakdown of the outer blood-retina barrier formed by the retinal pigment epithelium.^87^ OCT images of such areas demonstrate elevation of neurosensory retina by the presence of subretinal fluid (Figure 4). Sometimes this involves a visual acuity decrease that is correlated with the amount of liquid present in the layers of the sensory retina. Although, CSCR typically affects young men and has been described as a benign and self-limiting disease, it has a tendency to re-occur.^88^ Some eyes with CSCR may, however, have a poor visual outcome due to retinal pigment epithelium atrophy, persistent pigment epithelial detachment, subretinal fluid, recurrences, and submacular choroidal neovascularization.^89^ The precise pathophysiology of central serous chorioretinopathy is uncertain. It was originally thought to be a disorder of the RPE. Choroidal microcirculation abnormalities, possibly caused by atherosclerotic lesions, could play a role.^90^ Indeed, the disease could originate from choroidal hyperperfusion.^91–93^ Hp has been associated with the development of atherosclerosis^94^ and with the enhanced instability of atherosclerotic plaques.^95^ Moreover, cross-mimicking mechanisms between antibodies against Cag-A and vascular wall antigens could be involved.^95^ In addition, a cross-reaction between antibodies against anti-heat shock proteins and homologous host proteins has been proposed.^96,97^ It should be noted that even this theory of “molecular mimicry” is not completely supported. CSCR patients often have higher levels of serum and urinary cortisol and catecholamines than healthy subjects.^98,99^ Hypercoagulability and the enhanced platelet aggregation have been described in patients with CSCR^100^ and could somehow affect the choroidal circulation and increase its permeability. Furthermore, even if its role is unclear, the platelet-derived growth factor contributes to the pathogenesis of CSCR.^101^ Usually, the main risk factors for the onset of CSCR are glucocorticoids.^102^ These hormones increase platelet aggregation and blood viscosity^103^ and are capable of producing a vasoconstrictive response.^104^ These factors together would reduce the vascular bed, but on the other hand, they could lead to hypoperfusion and an increase in endoluminal perfusion pressure, leading to serum leakage and the presence of small molecules in the retina.^105^ Nevertheless, although several potentially associated risk factors for this disease have been reported (Table 1) and numerous studies have been conducted on this disease over the years, many aspects of CSCR remain unclear. Moreover, a correlation between CSCR and Hp infection has been suggested.^128–130^ A possible point of contact between these 2 diseases may resemble the interaction between Hp infection and atherosclerosis. Indeed, chronic infection with Hp may be involved in the development of the atherosclerosis via endothelial dysfunction and systemic and vascular inflammation,^131^ even if more recent studies exclude this association.^132,133^ It is still interesting to note that anti-Hp treatment can produce the faster reabsorption of the subretinal fluid.^134^ Dang et al^135^ suggest that *Hp* eradication could increase central retinal sensitivity. It must be remembered, however, that the metronidazole used in Hp eradication therapy improves intestinal microcirculation in septic rats independently of the bacterial burden, causing a significant improvement in their functional capillary density.^136^ Furthermore, antibiotic treatment significantly reduced adverse cardiac events in patients with acute coronary syndromes; however, this effect was independent of Hp seropositivity.^137^

**TABLE 1:**
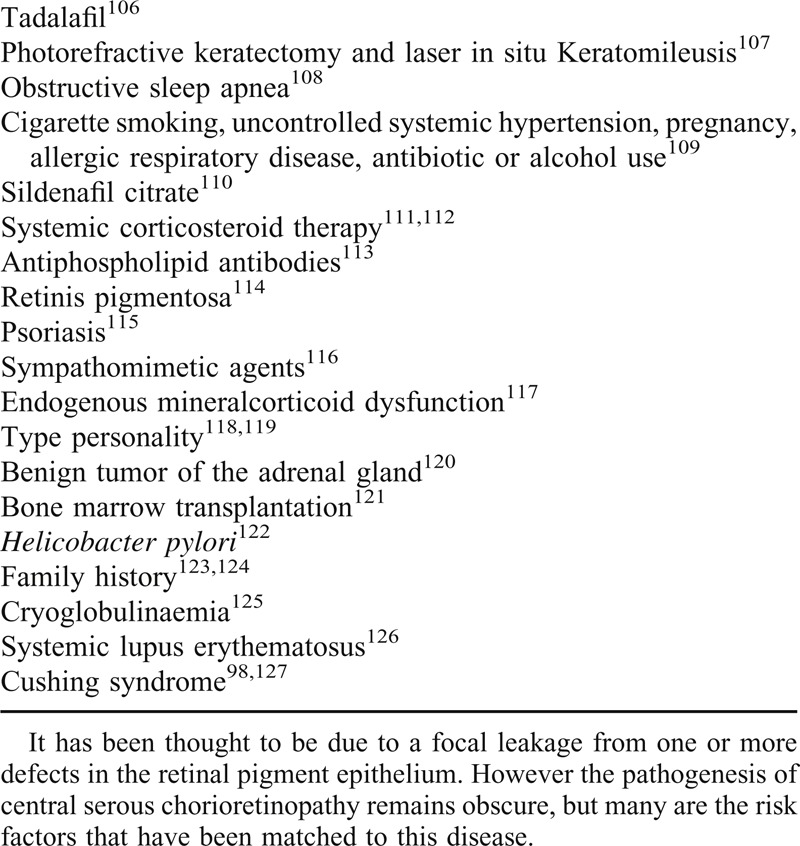
Potentially Risk Factors in Central Serous Chorioretinopathy

### *Helicobacter pylori* and Ocular Adnexal MALT Lymphoma

**FIGURE 5:**
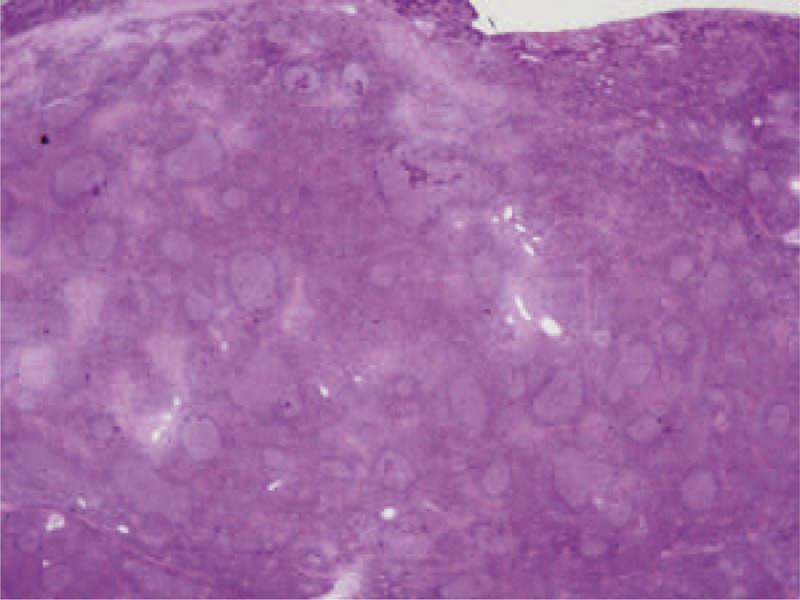
Extranodal marginal zone B-cell lymphoma of mucosa-associated lymphoid tissue (MALT lymphoma): the lymphoma cells infiltrate around reactive B-cell follicles, external to a preserved follicle mantle, in a marginal zone distribution. Marginal zone B cells have characteristics of small and medium size. The majority of patients present with stage I or II disease. The MALT lymphoma and its natural course is indolent and is slow to disseminate; recurrence may involve other extranodal sites.

MALT lymphoma is a form of lymphoma involving the Mucosa-Associated Lymphoid Tissue (MALT) that has distinct features from all other forms of primary non-Hodgkin extranodal lymphoma (Figure 5). Ocular adnexal lymphoma is primarily found in older adults with a slight female preponderance. It occurs in the orbit, conjunctiva, and lacrimal gland, in decreasing order of frequency of involvement.^138^ From a histological point of view, this condition is characterized by a large prevalence of marginal zone B-cell histologic types and a varying degree of infiltrating reactive T-cells.^139^ The clinical manifestations depend on the identity of the compromised structures. For example, 25% of MALT lymphoma displays conjunctival involvement. Intra-orbital masses are present in 75% of cases, while bilateral involvement occurs in 10%–15% of cases.^138^ Intraorbital lymphoma is variably associated with exophthalmos, palpable mass or nodule, eyelid ptosis, diplopia, epiphora, and impaired ocular motility.^140^ Its clinical presentation usually consists of a single, slowly growing, painless mass that displaces the normal structures; however, its presentation can also be acute, with inflammatory-like signs and symptoms. Ocular infiltration is exceptional.^141^ Gastric MALT is known to be acquired in response to local infection by Hp, which is present in greater than 90% of these lymphomas.^142^ To colonize in the stomach, Hp must overcome the acidic environment of the stomach and then the gastric mucous layer. The hydrolysis of urea with the generation of ammonia may enable survival of this acid-sensitive organism in the gastric mucosa. Furthermore, ammonia generated by urea hydrolysis may also produce severe cytotoxic effects within the gastric epithelium.^143^ Due to the spiral shape and multiple polar flagella, which are used for motility,^144^ Hp stick out through the mucous layer and reach the gastric epithelium, where they sticks to its cells using the adhesins.^145^ Among infected individuals, approximately 10% develop peptic ulcer disease; 1% to 3% develop gastric adenocarcinoma; and <0.1% develop MALT lymphoma.^146^ The contact between Hp and the gastric epithelium generates an immune and inflammatory response.^147^ This activates the transcription factor NF-kB, inducing proinflammatory chemokines and recruiting neutrophils, monocytes, macrophages, and dendritic cells.^148–151^ Furthermore, this response regulates processes connected with B-cell development, growth, and survival by producing cytokines and growth factors and can also be responsible for activating cell apoptosis.^152^ Then, an adaptive immune response to the Hp infection emerges from the macrophages and dendritic cells located in the lamina propria of the gastric mucosa.^150,153^ Neutrophils, monocytes, and macrophages may phagocytose Hp, but they seem to be able to achieve this without intracellular killing,^154^ as Hp can survive within monocytes for up to 48 hours.^155^ The failure of the immune response to eliminate Hp results in chronic inflammation of the gastric mucosa. The sequential progression from chronic inflammation to mucosal atrophy, metaplasia, and dysplasia leads to carcinogenesis.^156^ In a small subset of individuals, chronic inflammation due to a persistent Hp infection can give rise to organized lymphoid tissue in the gastric mucosa, ultimately progressing to low-grade gastric B-cell lymphoma of the MALT type.^157^ Immunity to Hp and gastric immune-mediated damage is dependent on T cells.^158^ The prolonged interaction between the bacteria and the host immune mechanisms makes Hp a plausible infectious agent for triggering autoimmunity via molecular mimicry. The tumor cells of low-grade gastric MALT lymphoma (MALToma) are B cells that are still responsive to differentiation signals, such as cytokines produced by antigen-stimulated T cells, and that are dependent on stimulation by Hp-specific T cells for growth.^157,158^ The activity of these specific T cells, which are defective in both perforin- and Fas ligand-mediated cytotoxicity, consequently promotes both B-cell overgrowth and exhaustive B-cell proliferation.^159^ In the gastric mucosal cells, there are elevated levels of cytokines, including proliferation-inducing ligand (APRIL), which belongs to the tumor necrosis factor family. APRIL is produced by macrophages present in the gastric MALT infiltrate, close to the neoplastic cells,^160^ and may also induce B-cell transformation and their progression to diffuse large B-cell lymphoma. APRIL production by macrophages can be enhanced and maintained by activated T lymphocytes.^161^ The survival and transformation of B cells in malignant lymphoma require additional signals. They come either from T cells or directly from the antigenic autostimulation of lymphoma cells. Therefore, one or more neoplastic clones, derived from a gastric MALToma, are able to express molecules that powerfully stimulate B-cell activation and proliferation,^162^ colonize and replace the original follicles, eventually destroying the gastric glands to form lympho-epithelial lesions,^163^ and finally precipitate the onset of low-grade gastric MALT lymphoma. Although MALT lymphoma usually grows slowly and has a low propensity to spread, a small percentage of cases undergo high-grade transformations.

Many MALT lymphomas at non-ocular sites are associated with an infectious etiology, supporting the model of antigen-driven lymphoma genesis.^164^ According to this model, an infection first triggers the chronic antigen stimulation of B cells and the production of antibodies. The proliferation of B cell clones becomes antigen-independent, and with uncontrolled proliferation, malignant transformations can occur.^164^ However, not all those with an Hp infection also have a MALT lymphoma. This indicates that the role played by genetic factors, is of great importance, as gastric MALT lymphoma presents with a series of recurrent genomic lesions, including chromosomal translocations and unbalanced aberrations. The accumulation of genetic abnormalities is associated with a loss of dependency from antigenic stimulation (with subsequent antibiotic resistance) and a possible histologic transformation.^165^ Moreover, 10%–20% of patients do not respond to Hp eradication treatments. This group often has a chromosome translocation, which suggests that there is another pathogenetic mechanism of MALT lymphoma that is thus far unknown.^161^

A higher incidence of MALT lymphoma has been reported in patients with chronic Hp infections, as well as in those with Sjögren syndrome.^158^ Similarly, patients with Sjögren syndrome have a much higher incidence of developing lymphoma, most of which are MALT type.^166^ Further studies are needed before Hp can be implicated as a significant contributor to the etiology of conjunctival MALT lymphoma.

### *Helicobacter pylori* and Anterior Uveitis

Uveitis is a term used to describe different forms of intraocular inflammation involving the uveal tract of the eye; it is classified by anatomical location and time course of the disease.^167^ Acute anterior uveitis, also known as iridocyclitis or iritis, is an inflammatory disorder of the iris and/or pars plicata (anterior ciliary body) and anterior chamber that lasts no longer than 3 months. Intermediate uveitis, or pars planitis, consists of vitritis, defined as an inflammation of the cells in the vitreous, sometimes with snow banking, or the deposition of inflammatory material on the pars plana. Posterior uveitis indicates inflammation in the retina and/or choroid. Uveitis is a rare disease that is particularly prevalent in younger people.^168^ The etiological diagnosis of anterior uveitis can be established in approximately 60% of cases, while 75% of patients with intermediate uveitis remained without specific diagnosis. A specific diagnosis could be established in 78% of patients with posterior uveitis. Uveitis infections accounted for approximately 20% of the above cases.^169^ Otasevic et al^170^ have demonstrated that a high percentage of antibodies to Hp in the serum of a group of patients with acute anterior uveitis, some of whom were affected by spondyloarthropathies. Unfortunately, the sample examined was too small to allow for any concrete conclusions. Kim et al^171^ have detected on a sample of 165 subjects that Hp infection is associated with high IOP in anterior uveitis, but without finding a real causal connection between Hp infection and ocular hypertension. Thus, uveitis involved in a multitude of diseases; many chronic inflammatory diseases are associated with an elevated risk of uveitis, eg, rheumatoid arthritis, ankylosing spondylitis,^172^ Behcet disease,^173^ and Crohn disease.^174^ These diseases can induce other types of uveitis due to inflammation, even if they are not diagnosed as uveitis at the beginning. The breakdown of the blood-aqueous barrier in uveitis involves cellular infiltration, an increase in protein permeability, and the up-regulation of cytokines, such as TNF-α and IL-6, and chemokines, such as MCP-1, and MIP-1. In the aqueous humor and uveal regions,^175^ the exposure of the cells near the blood-aqueous barrier to inflammatory cytokines and chemokines could eventually cause cytotoxicity, leading to apoptosis or proliferation. Inflammation is associated with increased oxidative stress by elevated ROS, which could alter cellular and molecular targets and pathways crucial to normal tissue homeostasis.^176,177^ The generation of ROS in turn activates redox-sensitive transcription factors such as NF-κB, which controls the expression of a large number of genes involved in apoptosis, cell growth, survival, differentiation and immune response. Alterations in NF-κB activity are associated with a large number of diseases, including autoimmune, cancer and inflammatory diseases.^178^ NF-κB plays an important role in the regulation of immune and inflammatory responses. Pathogens, oxidants, cytokines, chemokines, and growth factors associated with oxidative stress trigger specific receptors and cause oxidative stress signaling cascades that lead to the activation of NF-κB. NF-κB activation is responsible for the expression of a wide variety of genes that encode cytokines (TNF, IL-1, IL-6), chemokines (MIP-1, MCP-1), adhesion molecules (ICAM, VCAM, E-Selectin), iNOS and Cox-2.^175^ ROS are important modulators of signaling pathways and can regulate both the apoptotic signaling and NF-kappaB transcription triggered by TNF. ROS can also cause redox modifications that inhibit NF-kappaB activation, leading to cell death triggered by TNF.^179^ The increased ROS levels during inflammation could be increased oxygen consumption in the uveitis or decreased antioxidative defense in the concerned tissue. The increased levels of ROS in the ocular cells cause a redox imbalance, leading to activation of redox signaling intermediates, which in turn activate transcription factors, including NF-κB, with the result of transcription of inflammatory marker genes.^180,181^ The reduced circulating levels of vitamin C in Hp infected subjects may contribute to the etiology of some diseases associated with antioxidant deficiency.^182^ The excessive ROS generation also weakens the tissue own antioxidant defense system, further aggravating the inflammation and ROS production and generating tissue damage in uveitis. This damage increases the level of metallo-proteases, which chew up intra-cellular and extracellular proteins, resulting in tissue injury.^176^ During Hp infection, activated macrophages produce the following pro-inflammatory cytokines: IL-1, IL-2, IL-6, IL-8, and TNF-α.^183^ Most of these cytokines are expressed in the aqueous humors of patients with idiopathic acute anterior uveitis.^184^ Therefore, the chance of an autoimmune reaction due to molecular mimicry may be possible.^185,186^

## CONCLUSION

In conclusion, it is extremely difficult to compare the results of the studies that are currently available in the literature, as to do so would require a population-based study involving thousands of patients in order to effectively determine the prevalence of eye diseases in patients infected with Hp. Nevertheless, inadequate antioxidant protection or excess production of ROS creates conditions of oxidative stress, which is thought to play an important role in the aging of the eye and in many inflammatory eye diseases.^5^ In any case, it is difficult to understand how Hp infection can be linked to such varied pathologies. It is possible that this “link” might be the oxidative damage that recurs in circulatory disorders,^187^ inflammation^188,189^; and glaucoma.^55,85^ As we can also see in central serous chorioretinopathy and ocular adnexal MALT lymphoma, the effects of oxidative stress can be substantial. Inflammation in Hp infections and in eye diseases progresses through a series of common pathogenic aspects shared by the two entities, despite their differing clinical features. Indeed, adequate antioxidant defenses responsible for scavenging free radicals are essential for redox homeostasis and inhibition of inflammation. These are variables, eg, corneal epithelial cells have strong antioxidant defenses, conversely, other ocular tissues, such as the trabecular meshwork, are poorly equipped with antioxidant defenses and consequently less able to counteract injurious effects of ROS^5^ Therefore, therapy with antioxidants should prove beneficial for the clinical management of patients with Hp infection.^190^

Vitamin C appears to have a particularly important role, in fact within the cell, vitamin C helps to protect membrane lipids from peroxidation by recycling vitamin E.^191^ This could be relevant in all eye diseases that we talked about, especially in glaucoma where the Vitamin C has a direct effect on the trabecular meshwork where might improve their ability to degrade proteins within the lysosomal compartment and recover tissue function.^192^ Therefore Ascorbic acid supplementation may improve the effectiveness of Hp-eradication therapy.^193^ Besides, diets rich in naturally occurring ascorbic acid are associated with protection of the gastric corpus from atrophy and a reduction in the incidence of gastric cancer possibly through the ability of ascorbic acid to reduce oxidative damage to the gastric mucosa by scavenging carcinogenic *N*-nitroso compounds and free radicals and attenuating the Hp-induced inflammatory cascade.^193^ Further studies are required to prove that Ascorbate or other antioxidant supplementations have a significant impact on progress of the association between eye diseases and HP infection.

On the whole, the data reported in this review provide evidence that oxidative stress and inflammation represent common pathogenic mechanisms that play a major role in both Hp infection and several eye diseases.
